# New Amniotic Membrane Based Biocomposite for Future Application in Reconstructive Urology

**DOI:** 10.1371/journal.pone.0146012

**Published:** 2016-01-14

**Authors:** Jan Adamowicz, Marta Pokrywczyńska, Jakub Tworkiewicz, Tomasz Kowalczyk, Shane V. van Breda, Dominik Tyloch, Tomasz Kloskowski, Magda Bodnar, Joanna Skopinska-Wisniewska, Andrzej Marszałek, Malgorzata Frontczak-Baniewicz, Tomasz A. Kowalewski, Tomasz Drewa

**Affiliations:** 1 Chair of Urology, Department of Regenerative Medicine, Nicolaus Copernicus University in Torun, Ludwik Rydygier Medical College in Bydgoszcz, Bydgoszcz, Poland; 2 Department of General, Oncologic and Pediatric Urology, Nicolaus Copernicus University, Bydgoszcz, Poland; 3 Department of Urology, Nicolaus Copernicus Hospital Batory, Torun, Poland; 4 Laboratory of Modeling in Biology and Medicine, Institute of Fundamental Technological Research, Polish Academy of Sciences, Warsaw, Poland; 5 Department of Internal Medicine, Division of Infectious Diseases, University of Pretoria, Pretoria, South Africa; 6 Department of Clinical Pathomorphology, Faculty of Medicine, Nicolaus Copernicus University, Bydgoszcz, Poland; 7 Department of Chemistry of Biomaterials and Cosmetics, Faculty of Chemistry, Nicolaus Copernicus University, Nicolaus Copernicus University, Bydgoszcz, Poland; 8 Electron Microscopy Platform, Mossakowski Medical Research Centre, Polish Academy of Sciences, Warsaw; 9 Department of Mechanics and Physics of Fluids, Institute of Fundamental Technological Research, Polish Academy of Sciences, Warsaw, Poland, Poland; Peking Union Medical College Hospital, CHINA

## Abstract

**Objective:**

Due to the capacity of the amniotic membrane (Am) to support re-epithelisation and inhibit scar formation, Am has a potential to become a considerable asset for reconstructive urology i.e., reconstruction of ureters and urethrae. The application of Am in reconstructive urology is limited due to a poor mechanical characteristic. Am reinforcement with electrospun nanofibers offers a new strategy to improve Am mechanical resistance, without affecting its unique bioactivity profile. This study evaluated biocomposite material composed of Am and nanofibers as a graft for urinary bladder augmentation in a rat model.

**Material and Methods:**

Sandwich-structured biocomposite material was constructed from frozen Am and covered on both sides with two-layered membranes prepared from electrospun poly-(L-lactide-co-E-caprolactone) (PLCL). Wistar rats underwent hemicystectomy and bladder augmentation with the biocomposite material.

**Results:**

Immunohistohemical analysis (hematoxylin and eosin [H&E], anti-smoothelin and Masson’s trichrome staining [TRI]) revealed effective regeneration of the urothelial and smooth muscle layers. Anti-smoothelin staining confirmed the presence of contractile smooth muscle within a new bladder wall. Sandwich-structured biocomposite graft material was designed to regenerate the urinary bladder wall, fulfilling the requirements for normal bladder tension, contraction, elasticity and compliance. Mechanical evaluation of regenerated bladder wall conducted based on Young’s elastic modulus reflected changes in the histological remodeling of the augmented part of the bladder. The structure of the biocomposite material made it possible to deliver an intact Am to the area for regeneration. An unmodified Am surface supported regeneration of the urinary bladder wall and the PLCL membranes did not disturb the regeneration process.

**Conclusions:**

Am reinforcement with electrospun nanofibers offers a new strategy to improve Am mechanical resistance without affecting its unique bioactivity profile.

## Introduction

Every year, thousands of advanced surgical procedures are performed to replace or repair ureters, urinary bladders or urethrae that are damaged through disease or trauma. Well-established procedures of reconstructive urology utilise the small intestine for ureteral reconstruction or urostomy and continent urinary diversion [1, 2]. In addition, autografts derived from buccal mucosa and foreskin have an application in urethroplasty [3]. Although these sophisticated surgical techniques restore the proper function of reconstructed urinary tracts, they may increase the risk of stricture and fistula formation, and the development of metabolic disorders. An effort of regenerative medicine is to search for new biomaterials that are suitable for modern reconstructive urology, using the principles of tissue engineering [4].

Since the announcement of *in vitro* urinary bladder reconstruction by Atala et al., interest in urinary bladder wall augmentation has increased [5]. Despite promising results, this milestone in regenerative medicine has not been translated into clinical practice. Phase II studies conducted in children and adolescents with spina bifida showed lack of bladder compliance or capacity improvements after urinary bladder augmentation with an autologous cell seeded biodegradable scaffold [6]. Disregarding this unsatisfactory functional characteristic, the phase II study proved the feasibility of using an artificially fabricated material for human urinary bladder replacement at long-term follow-up. This is encouragement for us to look for new technologies and biomaterials that may be used for the reconstruction of urinary tracts.

Am have been used in medicine for more than 100 years. Am was first applied by Davis in 1910 for severe skin burns and management of hard-to-heal wounds [7]. Further studies on Am confirmed its unique properties i.e., anti-inflammatory and anti-scarring effects [8]. These observations led to frozen Am being used as a biological wound dressing in ophthalmology [9]. Since the 1960s, allo-implantation of Am has become a gold standard therapy for intractable epithelial defects, chemical and thermal burns, pterygium and persistent corneal ulcers, partial limbal cell deficiencies, ocular cicatricial pemphigoid, and Stevens-Johnson syndrome [10,11,12]. Allo-implantation of Am meets the expectation of scarless ocular surface healing for oculists; urologists should thus pay more attention to the characteristics of Am during reconstructive procedures.

Despite the appealing biocompatibility and bioactivity of Am, its low mechanical strength may discourage urologists from applying Am for augmentation of urinary tracts. Reinforcement of Am with electrospun nanofibers is a promising strategy to create novel biocomposite materials with attractive features that meet the necessary requirements for reconstructive urology.

In this study, we introduced design and *in vivo* biological evaluation of a biocomposite material composed of Am and electrospun PLCL nanofibers. This novel biocomposite material was used to replace the urinary bladder wall after partial cystectomy in a rat model.

## Materials and Methods

### Graft preparation

The sandwich-structured biocomposite material was constructed from frozen human Am (Eye Tissue Bank, Lublin, Poland) and covered from both sides with a two-layered PLCL membrane. Each PLCL membrane was prepared from the copolymer, PLCL7015 poly(L-lactide-*co*-ε-caprolactone) (Purac-Corbion, NL) containig 70:30 monomer units The electrospinning solution was made of 9% polymer dissolved in a mixture of solvents (chloroform+dimethylformamide, mass proportions 16:1). The electric potential was 15 kV, the solution throughput was 0.500 μl/h and spinneret to target distance was 20 cm.

PLCL is approved by the US Food and Drug Administration for medical applications, including body implants. Frozen pieces of 5x5 mm Am preserved in glycerol were sandwiched between the PLCL layers. Two consecutive initial layers of the PLCL nanofibers were used as a substrate to apply pieces of Am. An additional two layers of PLCL nanofibers were applied to the initial PLCL nanofibers layers containing Am in order to obtain a ‘sandwich-structure’. The orientation of the consecutive PLCL nanofibers were perpendicular to each other. The biocomposite material was cut to form patches (10x10 mm) with a ca. 2.5 mm margin left on the edge of the Am piece. Scanning Electron Microscopy (SEM) analysis of electrospun PLCL nanofibres containing Am was also conducted.

### Cell culture and PLCL membranes cytotoxicity test

Mesenchymal Stem Cells (MSC) were isolated from the bone marrow of rat femurs. Method of bone marrow derived MSC isolation was presented earlier [13]. Briefly, after the dissection of rat femurs, the obtained cell suspension from flushed bone marrow was centrifuged twice. Next, the cell pellet was suspended in fresh culture media and seeded in culture plates (BD Bioscience, USA). Cells were cultured in DMEM/Ham’s F12 media (Sigma, Germany) containing 10% fetal bovine serum (FBS, Sigma, Germany) supplemented with 5 μg/mL amphotericin B, 100 μg/mL streptomycin, 100 U/mL penicillin (Sigma, Germany) and 10 ng/mL basic fibroblast growth factor (bFGF, GIBCO, USA). Scaffolds of the biocomposite material were placed in culture plates and 6 x 10^6^ MSC were detached and seeded onto the scaffolds (n = 5) in triplicate at 1h intervals. The seeding procedure was repeated on the fourth and sixth day of culture. Cells were cultured on the scaffold surfaces for 7–14 days. MSC survival on PLCL membranes were analysed by SEM.

### Am Cytotoxic assay

Evaluation of Am cytotoxicity was conducted based on extract toxicity assay according to ISO-10993 norm. Cytotoxicity of AM extracts were evaluated using MTT Cell Proliferation assay and real-time cell analyser (xCELLigence RTCA DP, Roche Applied Science, Germany). Am (∼6 cm^2^) was extracted in DMEM/Ham’s F12 media (Sigma, Germany) containing 10% FBS (Sigma, Germany), 10 ng/ml bFGF (GIBCO, USA), 5 μg/mL amphotericin B (Sigma, Germany), 100 μg/mL streptomycin (Sigma, Germany) and 100 U/mL penicillin (Sigma, Germany) for 24 h (36°C, 5% CO_2_). After 24 h exposure to different concentrations (100%, 50%, 25% and 12,5%) of Am extract the number of live cells per well was determined by absorbance measurement at 570 nm. MSC viability was presented on histogram as an average from five measurements. To evaluate cytotoxicity of Am extracts using a real-time cell analyser MSC were seeded on E-Plates 16 (5×10^3^ cells/well) and cultured until reaching a log- phase growth in standard medium. After 24 h incubation, the same concentration of Am extracts were added to the wells. Cells were exposed to Am extracts for 72 h. Results were presented on a graph as an average from five measurements.

### Animals and surgical procedure

Ten-week old Wistar rats (n = 20) from one strain were selected for this study. All animals had comparable weights oscillating between 250–300g. Animals were anaesthetised with intraperitoneal sodium pentobarbitone using a dose of 50 mg/kg based on body weight. Cystoplasty was performed according to a previously described procedure [13]. Briefly, rats underwent hemicystectomy and their bladders were augmented with prepared biocomposite patches anastomosed using a 6–0 absorbable polyglycolic sutures. After surgery, animals were separated into individual cages and post*-*operative analgesia with opiate based pain killers was provided. After 3 months, animals were sacrificed by CO2 overdose and the reconstructed urinary bladders were collected for histochemical analysis (n = 10) and mechanical analysis (n = 10). Ethical permission was obtained from the local committee for the study (Local Ethics Committee for the Experimental Studies on Animals, University of Science and Technology, Bydgoszcz, Poland No. 2/2014).

### Histological and immunohistochemical analysis

Tissue specimens were fixed in 10% (v/v) neutral (pH = 7) buffered formalin and embedded in paraffin. Cross-sections of the entire reconstructed segment were prepared. Histological analysis using H&E staining was performed. The connective tissue components and muscle layers were stained according to TRI protocol. Immunohistochemical staining using anti-smoothelin antibodies (R4A, Abcam, Great Brittan) was conducted to identify contractile smooth muscle fibres within the regenerated urinary bladder wall [14]. Briefly, tissue sections were incubated with primary anti-smoothelin antibodies (dilution 1:400). After rinsing, the sections were overlaid with peroxidase-conjugated anti-mouse secondary antibodies (EnVision/HRP anti Mouse; Dako, Denmark). Stained samples were analysed by two independent pathologists using light microscopy.

### Digital evaluation of smooth muscle content

Digital images of anti-smoothelin and TRI stained specimens (640x480 pixels) were used for quantitative evaluation of smooth muscle content within the reconstructed urinary bladder wall. ImageJ was used for digital image processing [14]. The smooth muscle coverage (%) was measured based on constructed histograms from the obtained images. Analysis was conducted by one pathologist and repeated 20 times for each augmented urinary bladder. The same methodology was applied to estimate the smooth muscle content in each control group, which consisted of 20 non-operated urinary bladders derived from 10-week old Wistar rats. Statistical analyses was performed using an one way analysis of variance (ANOVA). Comparison between smooth muscle content in control and study groups for each staining technique was performed using a Student’s t-test (p<0.05) (JMP^®^ Pro 11.0.0 (64 bit) software copyright © 2013 SAS institute Inc).

### Analysis of the mechanical properties of the reconstructed bladder wall

Tensile tests were conducted on a load frame of a servohydraulic material testing machine (MTS 242.01 actuator, Eden Prairie, USA). Specimens tested were derived from the reconstructed bladder wall and intact bladder wall (control group n = 10). All specimens were derived from the dome of the bladder. The specimens (10 mm length, 10 mm width) were mounted into flat grips with a gauge base of 10 mm. During the test, the specimen was longitudinally stretched at a rate of 0.3 mm/s until failure. The grip travel and specimen load were continuously measured over the test procedure with a precision force transducer (Interface, model 1500, measuring range 125 N, resolution 0.0625 N) and a MTS system linear variable differential transformer (measuring range 100 mm, resolution 0.01 mm). The Young’s elastic modulus (MPa) was estimated based on generated Stress/strain curves. The same protocol was applied to measure Young’s elastic modulus of Am and PLCL.

## Results

### Ultrastructural analysis of scaffold

**Fig 1:**
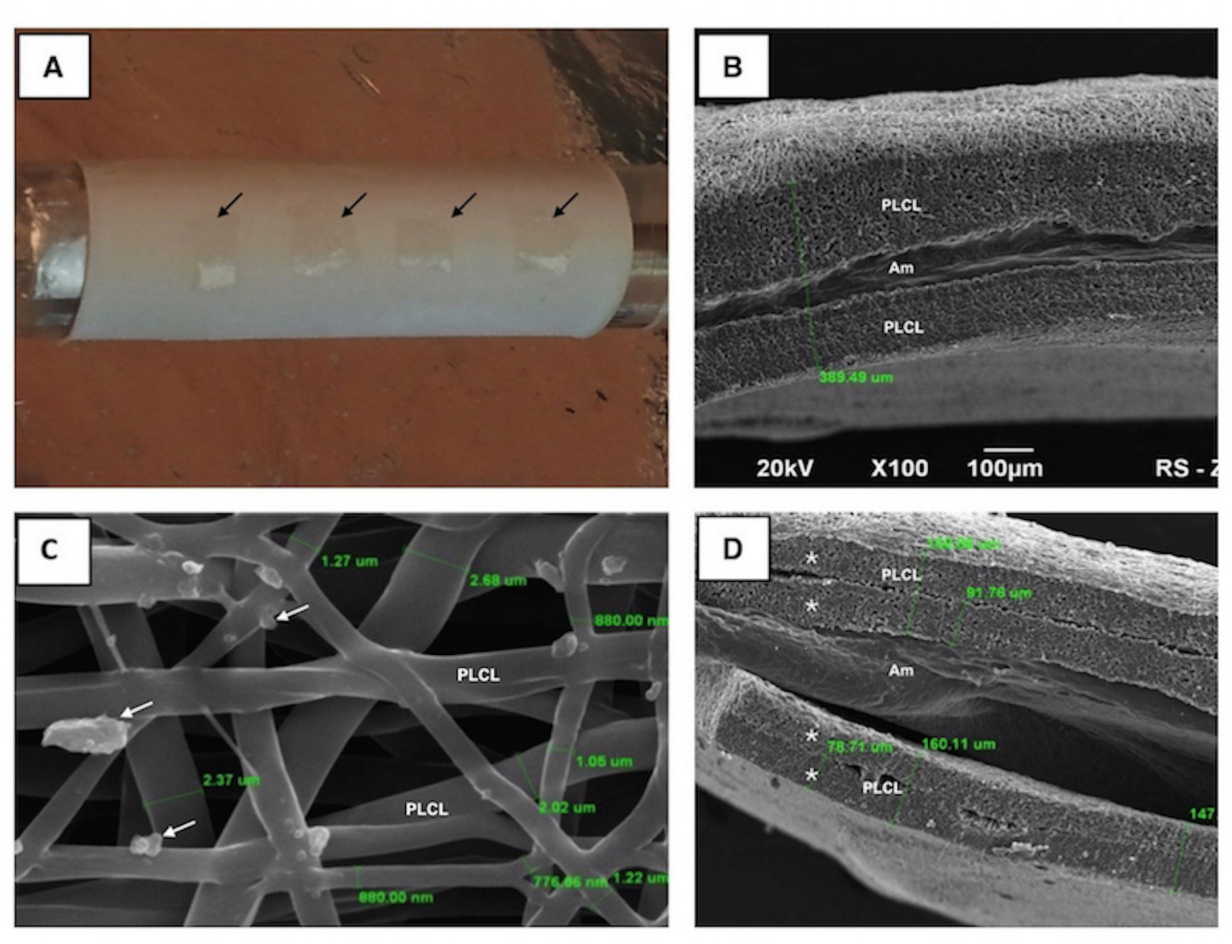
Preparation and structure of biocomposite. (A) The pieces of Am (black arrows) placed onto a sheet of PLCL nanofibers. A drum is used as a target during the nanofiber production process. SEM images are displayed in B-D. (B) A cross-section image of the biocomposite material. The biocomposite material is 389 um thick with an inner cavity containing the Am. (C) Visible drops of glycerin used for Am preservation are observed on surface of PLCL nanofibers (white arrows). (D) Two pieces of delaminated biocomposite material. The borders between consecutive sheets of nanofibers (*) are clearly visible with Am inside.

SEM examination revealed a clear line between the Am and PLCL layers, indicating a bilayer structure (Fig 1A, 1B and 1D). Fig 1D displays a delaminated piece of material where all layers of the created hybrid biocomposite material can be observed. Four layers of PLCL membranes (two on each side) with an inner cavity containing a thin Am can clearly be observed (Fig 1D). The analysed biocomposite material was approximately 400 μm thick (Fig 1D) and the diameter of the electrospun PLCL nanofibers ranged from 0.7–2.7 μm (Fig 1C).

### Cytotoxicity of PLCL membranes

**Fig 2:**
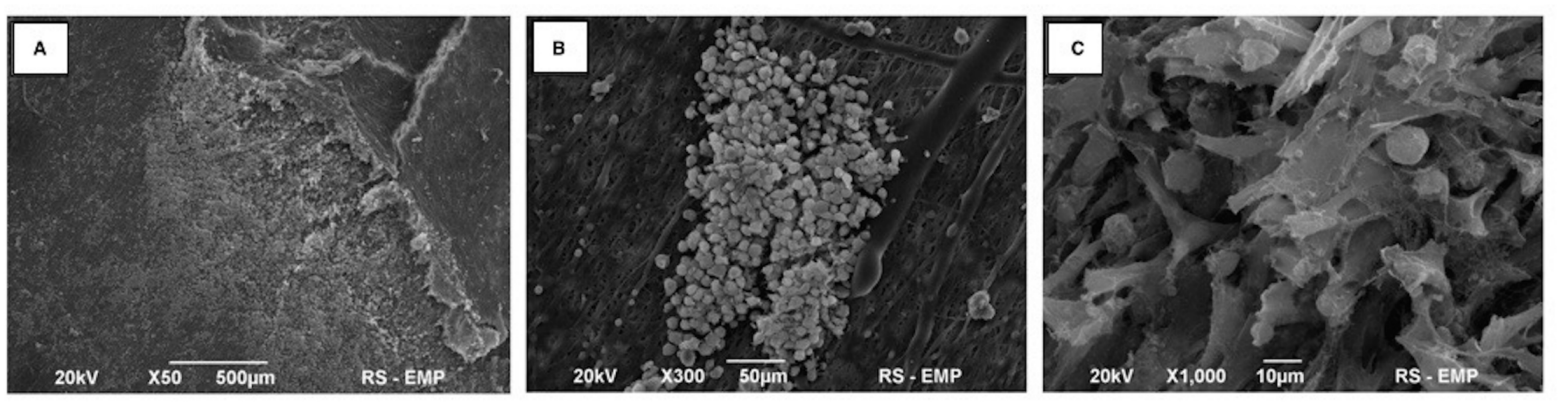
Cytotoxity testing of biocomposite using MSC. (A) MSC migrating towards Am on the 7^th^ day of *in vitro* cultivation. (B) Clusters of MSC distributed on an external surface of a PLCL membrane. (C) Dispersed MSC adherent to PLCL nanofibers.

MSC grew well on the PLCL membranes (Fig 2A–2C). After 7 days of incubation, large cell clusters covered the surface of the PLCL membranes (Fig 2A). Microscopic evaluation revealed extensive and deep cellular infiltration of the PLCL layers (Fig 2B and 2C). MSC tended to migrate towards the Am, gradually colonising its surface.

### Am Cytotoxic assay

**Fig 3:**
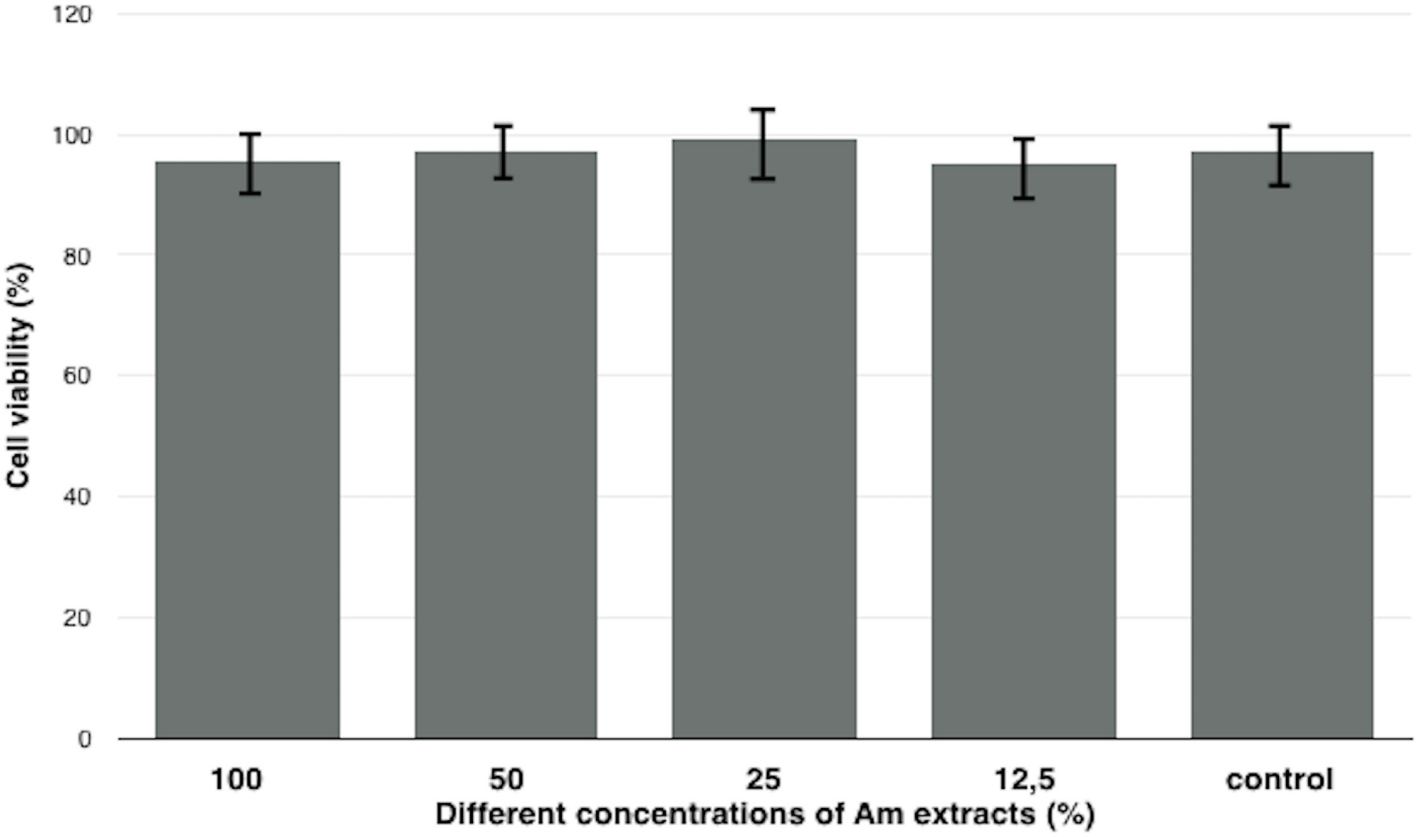
Amniotic membrane extract cytotoxicity measurement using the MTT assay. Each result was presented as an average from 5 independent experiment with SD bars. No statistically significant difference in cell viability was observed between AME treated and control cells (p>0.05) after 24 hours.

**Fig 4:**
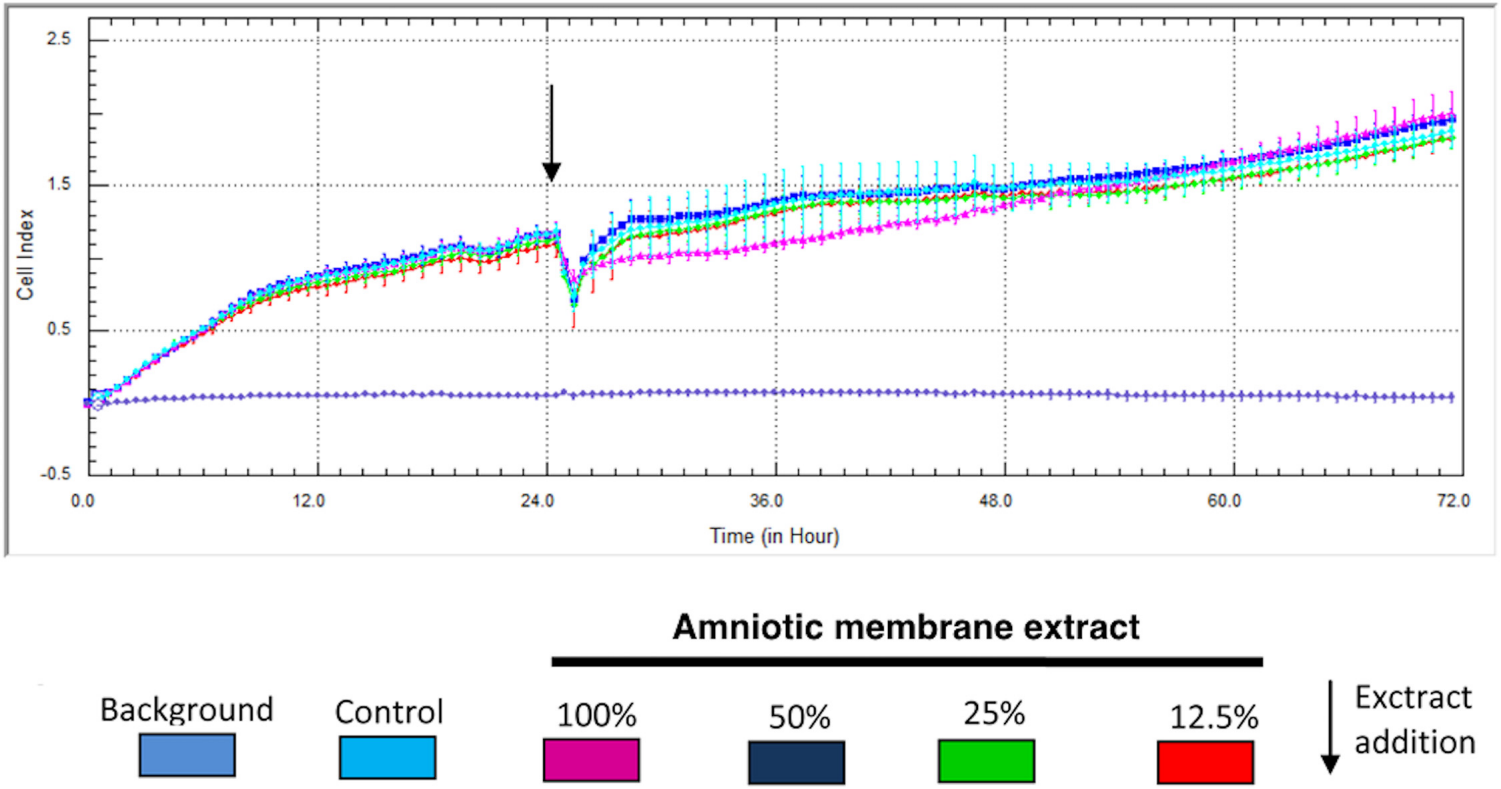
Amniotic membrane extract cytotoxicity measurement using real-time cell analysis. Each result was presented as mean from 5 independent experiment with SD bars. No statistically significant differences in cell viability were observed between AME treated and control cells (p>0.05) after 72 hours.

Both analysis, MTT and real-time cell analysis confirmed that the extract obtained from Am didn’t exhibit cytotoxic effects against adipose-derived porcine MSC (Figs 3 and 4). Am didn’t negatively influence the MSCs viability.

### Surgical procedure

**Fig 5:**
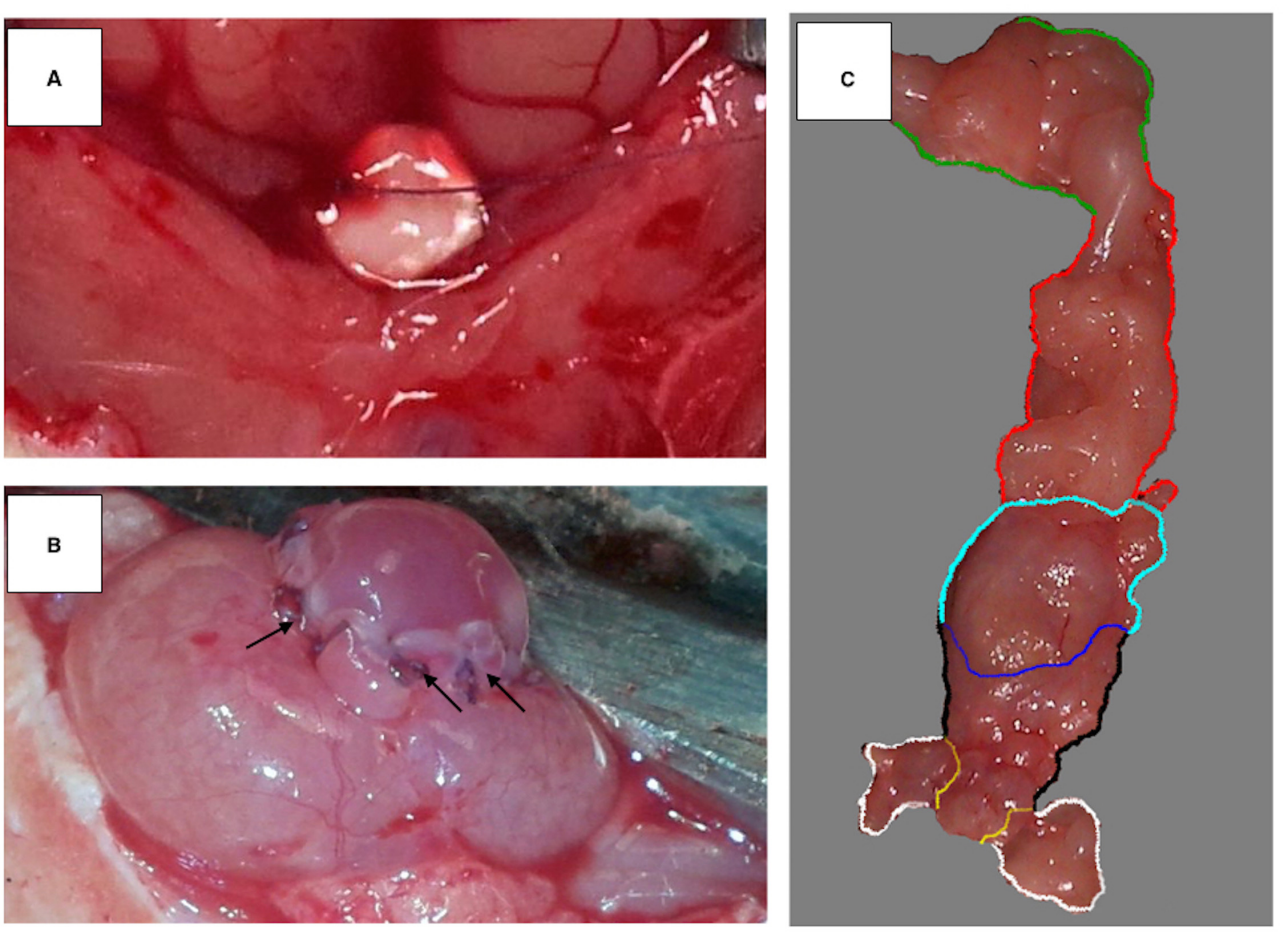
Urinary bladder augmented with biocomposite. (A) Biocomposite material scaffold prepared for the suture procedure. (B) Urinary bladder after the augmentation procedure. Single fixing sutures are visible (black arrows). The optimal compliance of the biocomposite material scaffold allowed for bladder filling shortly after the surgical procedure. (C) Resected reconstructed bladder 3 months after augmentation. The regenerated bladder wall (blue and cyan line was well integrated with the native bladder wall (black line). The borderline between the intact part of the bladder and the reconstructed one was indistinct and without scar formation (blue line). The upper surface of regenerated bladder wall (cyan line) was covered with adipose tissue forming a vascular pedicle (red line) derived from the omentum majus (green line). The bladder neck (yellow line) can be observed with adjacent fragments of seminal vesicles (white line).

All animals survived the surgical procedure. No side-effects were observed during follow-up. In all cases, no breakdown of the implanted patch or urinary leakage into the peritoneal cavity was observed. After 3 months of follow-up, the urinary bladder could easily be mobilised and resected (Fig 5A, 5B and 5C). The augmented urinary bladder wall was integrated with the host tissue. The suture line was not visible. Adhesions were mostly formed by the abdominal omentum majus. Macroscopic examination revealed rich vascularisation of the tissue engineered urinary bladder wall (Fig 5C).

### Histological analysis

**Fig 6:**
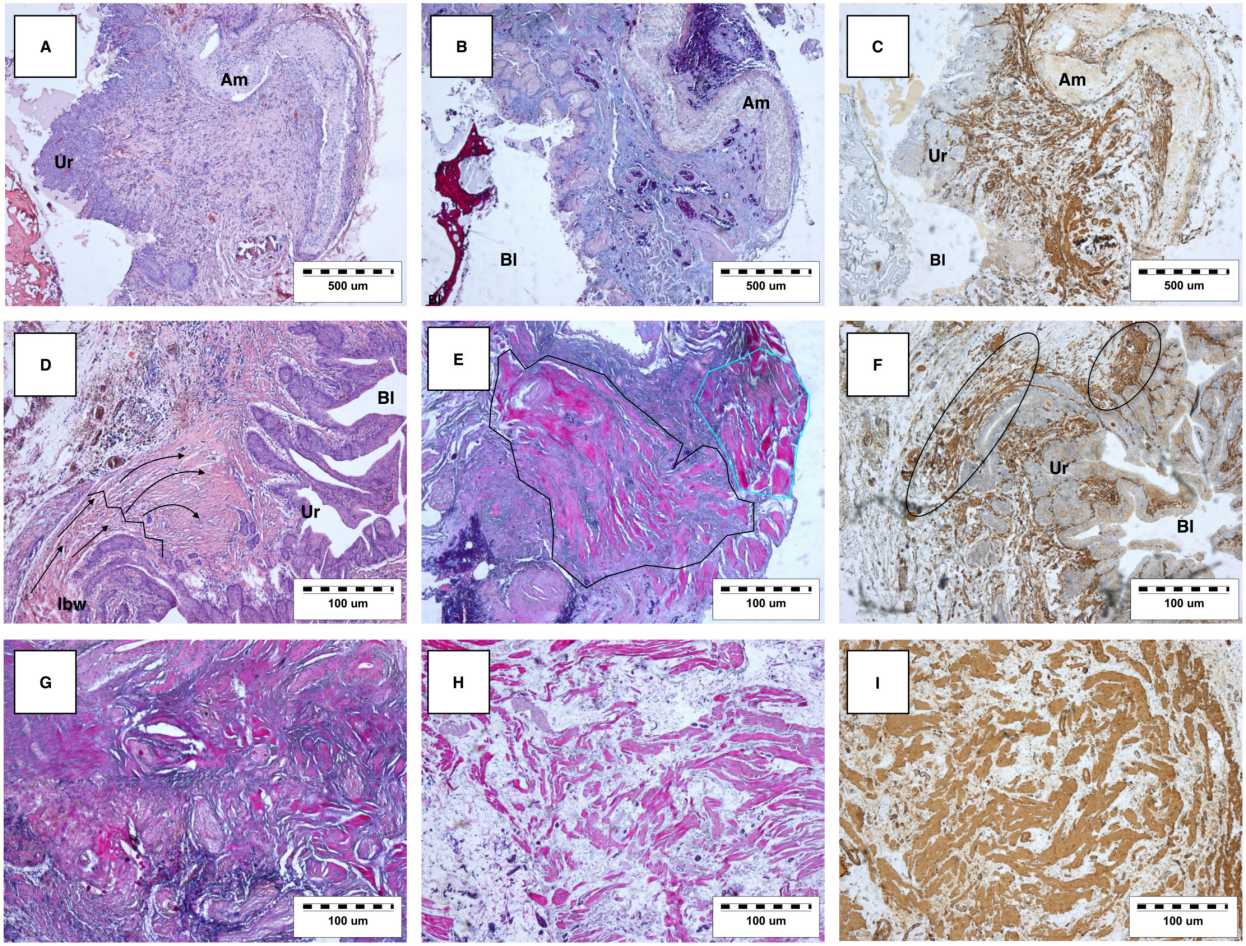
Histological and immunohistological analysis of the reconstructed urinary bladder wall. Am; Amniotic membrane, Ur; Urothelium, Bl; lumen of urinary bladder, IBW; Intact host urinary bladder wall. (A) H&E staining displaying mild inflammatory infiltration. (B) TRI displaying regenerating single muscle bundles from the central part of the reconstructed bladder wall. (C) Anti-smoothelin staining revealing frequently arranged smooth muscle bundles. Strong immunoreactivity beneath the urothelium layer is observed. (D) H&E staining revealing the border between the intact bladder wall and reconstructed bladder wall (zigzag line). The elongating smooth muscle cells (black arrows) gradually loose their layered architecture. Moderate inflammatory infiltration is also observed. (E) TRI displaying the regularly arranged smooth muscle bundles; some smooth muscle bundles run transversely (cyan line), but the most obvious bundles run longitudinally (black line). The specimen was obtained from the edge of the reconstructed bladder wall. (F) Anti-smoothelin staining displaying the distribution of smoothelin positive-cells (black ovals) under the urothelial layer. (G) TRI staining displaying the abundant disorganised hypertrophied smooth muscle bundles in the peripheral part of the reconstructed bladder wall. (H) TRI showing smooth muscle bundles separated by collagenous fibres in the central part of the reconstructed bladder wall. (I) Anti-smoothelin staining revealing abundant smoothelin expression in the peripheral part of the reconstructed bladder wall.

Cross-sections of the regenerated urinary bladder wall displayed a polarised structure with a reconstituted multilayered urothelium in all cases. Regrowing of the detrusor muscle was also observed. The Am acted as a surface for regenerating the urinary bladder wall. The partially preserved structure of the Am, incorporated into the new urinary bladder wall, was visible throughout the cross-sections. Am acted as a surface for growing neotissue, resembling a typical multilayered structure of the urinary bladder wall (Fig 6A, 6B and 6C). The urothelium covered the inner surface of the augmented urinary bladders and was hyperplastic. The lamina propria was almost completely collagenised with visible vessels.

Anti-smoothelin staining and TRI revealed bundles of regenerated smooth muscle; forming a muscular coat for the new urinary bladder wall. Immunohistochemical staining for smoothelin confirmed the presence of a terminally differentiated contractile phenotype, a characteristic of regenerated smooth muscle cells (Fig 6C, 6F and 6I). A rich distribution of smoothelin-positive cells were detected beneath the urothelium (Fig 6C and 6F). Bordering the intact and reconstructed urinary bladder wall, bundles rich in smooth muscle cells could be observed migrating and growing inwards (Fig 6D). The new muscular layer, located proximal to the intact part of the regenerated urinary bladder, was characterised with a regular arrangement of smooth muscle (Fig 6E). The reconstructed urinary bladder wall exhibited a predominant smooth muscle orientation in the longitudinal direction. In contrast to a normal urinary bladder wall, smooth muscle layers were significantly less distinct. The arrangement of the smooth muscle bundles became more irregular closer to the centre of the reconstructed urinary bladder wall. An uneven distribution pattern with an increase in abortive myocytes was observed, resulting in haphazardly organised smooth muscle bundles (Fig 6H). H&E staining of the reconstructed urinary bladder wall revealed a mild and moderate inflammatory infiltration (Fig 6A and 6D). The dispersed inflammatory infiltrate was determined to be composed mainly of lymphocytes. Degradation of the PLCL membranes was accomplished. No foreign-type remnants or multinucleated cells involved in the resorption of nanofibers were observed.

### Smooth muscle content

**Fig 7:**
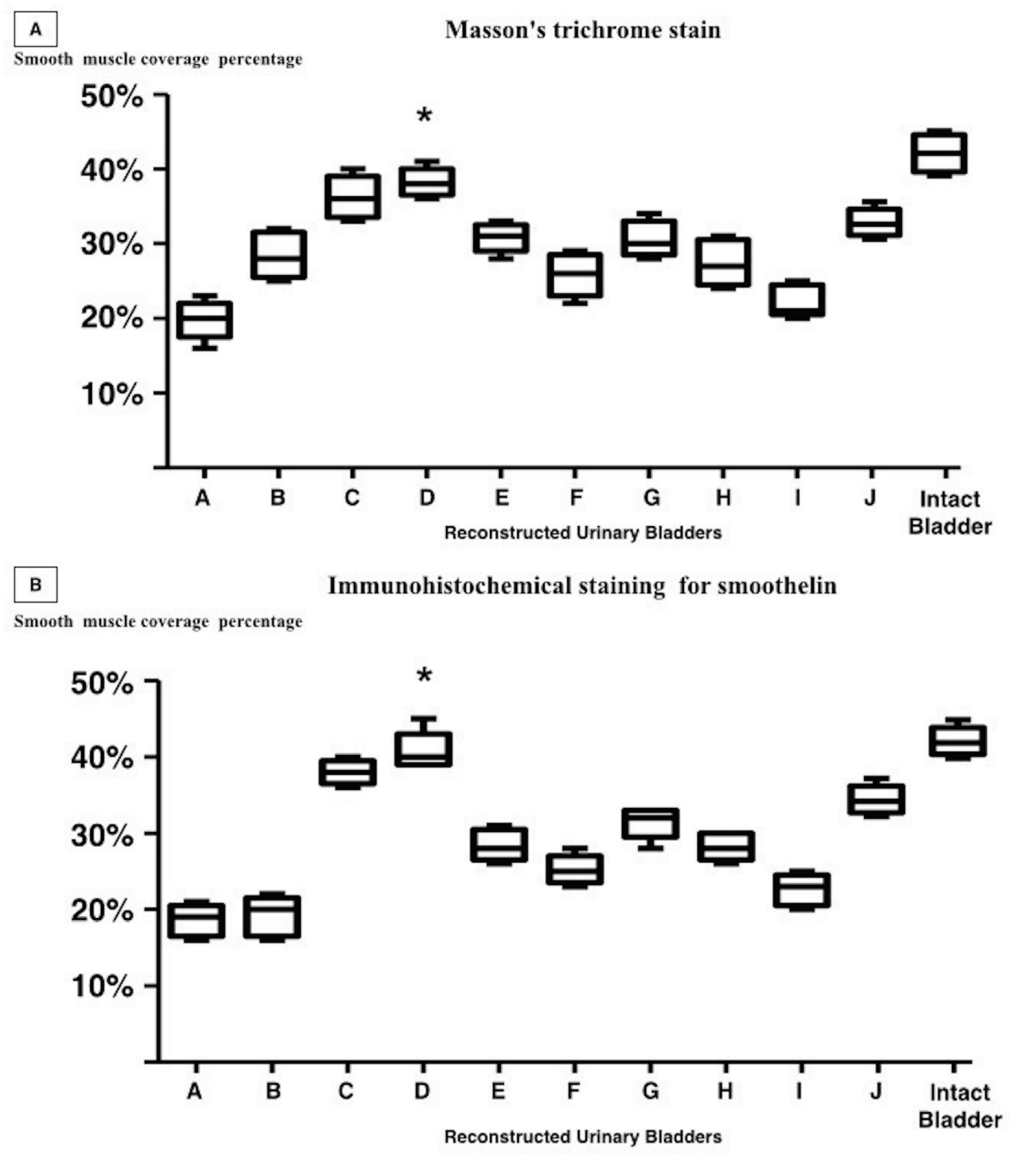
Percentage of the reconstructed bladder wall covered with smooth muscle. Staining with (A) TRI and (B) anti-smoothelin staining. The regenerated bladder wall with a statistically similar (TRI [p = 0.03] and anti-smoothelin staining [p = 0.35]) smooth muscle content compared to the bladder wall in the control group (*).

Anti-smoothelin staining and TRI determined smooth muscle coverage percentage of the specimens surface to be in range of 18.1% to 41.4% and 19.7% to 37.3% respectively (Fig 7A and 7B). Percentage of smooth muscle coverage in the control group was significantly higher (p ≤ 0.001) in the 9 augmented urinary bladders (anti-smoothelin; 41% coverage and TRI; 42% coverage). In one case, statistically significant similarity in smooth muscle coverage percentage was determined (Fig 7A and 7B sample D) (TRI [p = 0.03] and anti-smoothelin staining [p = 0.35]).

### Analysis of the mechanical properties of the reconstructed bladder wall

**Fig 8:**
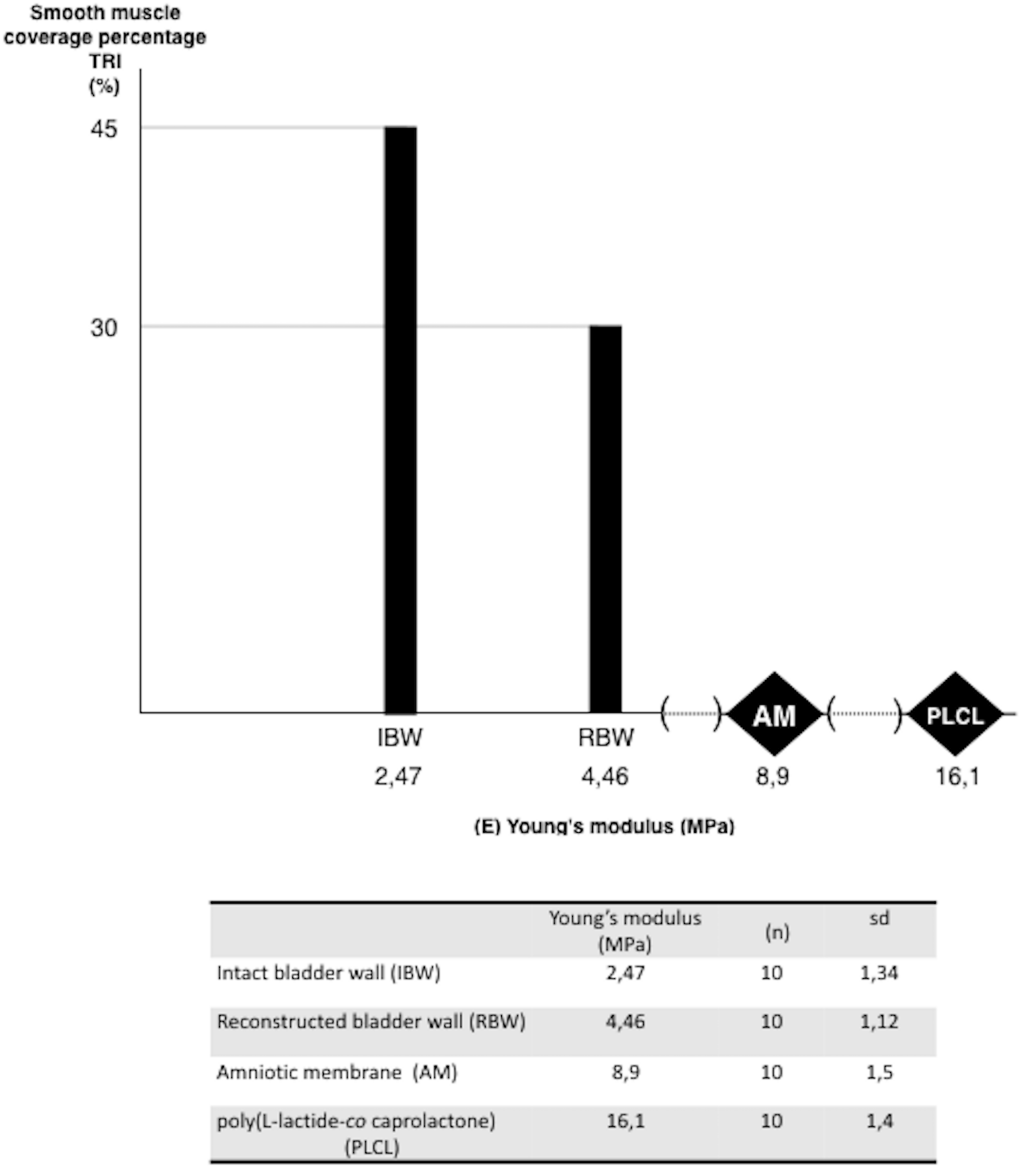
The mechanical evaluation of reconstructed bladder wall based on Young’s elastic modulus. Young’s modulus of intact and reconstructed bladder walls were compared to the digitally estimated content of smooth muscle content based on TRI staining average. Additionally, to reflect changes in the remodeling of the augmented bladder wall, Young’s modulus of Am and PLCL are presented. The presented values of smooth muscle content was rounded up.

In all cases Young’s modulus of the regenerated bladder wall was significantly higher (p<0.05) than the native bladder wall (Fig 8). This result indicates that the reconstructed bladder wall was stiffer in comparison to the native one. Mechanical evaluation of the reconstructed bladders based on Young’s modulus corresponded to the histological findings that confirmed the presence of fibrotic lesions and locally disordered detrusor cytoarchitecture within the augmented wall. Higher elasticity of the reconstructed bladder than Am alone indicates regeneration of the smooth muscle layer and components of the extracellular matrix that contribute to normal biomechanical properties of the urinary bladder wall. Smooth muscle content, which mainly influences biomechanics, was higher in the regenerated bladder wall that exhibited a lower Young’s modulus.

## Discussion

Extracellular matrix (ECM) of Am is composed of collagen (type I, III, IV, V and VI) fibronectin, nidogen, laminin, proteoglycans and hyaluronan in a proportion similar to the basement membrane of urinary tracts [15,16,17,18]. Multiple soluble active growth factors have been identified within cryopreserved Am [19]. This naturally derived composition of incorporated growth factors are predisposed to support fetal healing that is fundamentally different to that of healing in adults [20,21]. Wound healing in a fetus occurs rapidly via a regenerative process; without an inflammatory response, resulting in a complete restitution of normal tissue function [22, 23]. The ECM of Am develops within 8 days after fertilisation and belongs to an universal fetal systemic mechanism that regulates tissue response to damage or injury [24, 25, 26]. Longaker et al. demonstrated that the scarless healing properties of fetal skin are intrinsic to the fetal ECM and are not due to the fetal environment per se [27].

Am allografts could initiate scarless and ‘fetal-like’ healing in different types of adult tissue [28, 29, 30, 31, 32, 33, 34]. Am employed as a biological wound dressing reduced inflammation and minimised scarring in corneal pathology [35]. These reported therapeutical effects were linked to the synergistic action of diffusible growth factors released to the ocular surface. Following this, Güneş et al. postulated that covering a reconstructed section of an urethra after buccal mucosa graft urethroplasty would prevent postoperative restenosis and fistula formation [36].

Biotechnology offers strategies to enrich biomaterials for tissue regeneration with growth factors in order to customise their bioactivity spectrum and biocompatibility. Nevertheless, the usefulness of this approach is limited by insufficient research data explaining the intercellular dialog of multiphase processes like tissue regeneration [37]. In this situation, it is challenging to predict an *in vivo* tissue response on an artificially composed set of incorporated growth factors. A seemingly appropriate combination might trigger an unexpected adverse reaction. Nuninga et al. demonstrated that type I collagen biomatrices enriched with heparin-binding vascular endothelial growth factor, fibroblast growth factor and epidermal growth factor induced fibrosis and narrowing of the reconstructed urethra in a rabbit model [38]. Reproducing a biomaterial with a similar bioactivity potential as Am is difficult through current biotechnological processes. Application of Am for tissue engineering based on strategies for partial urinary tract replacement could guarantee a regeneration-enhancing effect, independent of growth factor enrichment or pre-seeding of cells.

Our sandwich-structured biocomposite material contained Am with an unmodified surface. This concept aimed to provide a high retention of active agents and preservation determined by their bioactivity and biocompatibility. The glycerine solution used as a preservation fluid formed a thin film that acted as a barrier during nano-spinning. Many cross-linking agents were recently evaluated to the increase strength and durability of Am [39, 40, 41, 42]. Nevertheless, this approach significantly influenced the biochemical composition and physical properties of Am, potentially affecting its clinical efficacy in urinary tract reconstruction[43].

Adequate biomaterial for reconstruction of urethrae, urinary bladders and ureters should be endurable but continuous on low volume changes, lacking the tendency to collapse or tear during the acute phase after implantation, to avoid urine leakage, and formation of strictures or fistulae [44]. During the patients daily activities, the biomaterial must withstand the forces exerted on it by pelvic musculature during neotissue growth [45]. Copolymers are found to be very useful during cellular growth due to their elastic behaviour and mechanical strength. We managed to obtain a flexible, elastic and tear-resistant membrane from PLCL composed of 70% L-lactide and 30% E-caprolactone.

The structure of the biocomposite material combines the bioactivity of Am, the synthetically produced durability and mechanical resistance of PLCL nanofibers that formed the elastic three dimensional frame, guaranteeing the necessary strength, shape and protection of Am. Am turned out to be an unsuitable material for urinary bladder reconstruction in our rat model due to its low mechanical resistance. We noticed the tendency of Am to tear during the bladder filling phase shortly after completed urinary bladder augmentation (unpublished data). PLCL nanofibers used in the biocomposite material had reinforced Am before the reconstructed bladder wall gained mechanical endurance resulting from gradual stroma development. No rupturing of the urinary bladder wall augmented with the biocomposite material was observed.

Fabricated PLCL layers gradually degraded within 8–10 weeks after implantation. The degradation-time of PLCL turned out to be optimal for cellularisation of Am and neotissue formation in our rat model. It is unknown if such a rate of PLCL scaffold degradation would be optimal for reconstructive urology and should be investigated in the near future. The degradation rate of PLCL scaffolds was determined to be faster *in vivo* than *in vitro* [46]. By changing the monomer content of PLCL copolymers; degradation behaviours and mechanical properties can be adjusted [47,48]. Our research revealed that the PLCL scaffold exhibited eight fold more stiffness compared to native rat bladder tissue. Taking this physical property of PLCL in to account, natural distension and contraction would be impossible if the complete PLCL degradation of nanofibers didn't occur. Mechanical mismatching is a major obstacle in designing a scaffold for bladder tissue engineering applications.

Partial cystectomy in a rat model revealed regeneration of the urinary bladder wall including complete re-epithelialisation and reconstitution of the muscular layer within 12 to 14 weeks [49]. Between the second and third week after implantation, contractile tissue started to add traction to the matrix scaffold, leading to shrinkage of the implant and formation of fibriotic barriers. This limited the accessibility of the scaffolds to slow elongating muscles bundles and sprouting nerves [50]. The role of the PLCL layers was to keep Am in a flattened arrangement during the first phase of remodelling to facilitate its colonisation. We believe the stable spatial environment provided by the biocomposite enabled restoration of reconstructed urinary bladder wall layered structure observed in our study.

Biomaterial used for urinary tract reconstruction should have physical properties making it convenient for implantation during surgical procedures and allow for it to constitute a reproducible surgical technique. The usage of Am in ophthalmology displayed susceptibility to ruptures propagating from areas traumatised during suturing [51]. Our prepared biocomposite material could easily be twisted and bent. In addition, fixing sutures can be placed along the edge of the tear-resistant nanofiber layer. Polymeric scaffolds play a crucial role in the process of engineering new tissues, effecting cellular growth and viability. In addition to improving the mechanical properties of Am, the role of the PLCL membranes were to promote cellularisation of Am by the host’s cells. A propagating migration of MSC towards the Am was observed during the MSC cultivation on biocomposite. Local injury-activated cells (including urothelial and smooth muscle cells) expressed a similar migratory mechanism to that of MSC [52, 53, 54, 55, 56, 57,58]. PLCL nanofibers provided good adhesion sites and stem cells could migrate perpendicularly along the nanofibers. The regular orientation of electrospun PLCL nanofibers aimed to support graft cellularisation and restoration of the neotissue's three dimensional structure.

One of our previous studies compared scaffolds prepared from PLCL alone and decellularised aortic arch to be used as a ureter replacement using a rat model [59]. PLCL scaffolds appeared to be a better template for regeneration in terms of smooth muscle regrowth and restoration of a new ureter wall with a layered histo-structure. Saratonev et al. conducted an interesting comparison based on the cytokeratin expression pattern for differentiated and stratified human urothelium between PLCL membranes and Am as cellular matrices for urothelial cells [60]. PLCL membranes supported the urothelium proliferation significantly more than Am. Considering this observation, PLCL layers improve re-epithelialisation of Am at the initial stage of urinary tract regeneration. Surprisingly, our earlier results indicated that a scaffold made from PLCL alone was not suitable for rat urinary bladder augmentation due to severe fibrosis and lack of smooth muscle regeneration [44]. This result in relation to effective urinary bladder augmentation with biocomposite material underlies the importance of Am during the regenerative process.

The concept of applying Am in reconstructive urology was introduced for the first time by Lenko et al. in 1955 [61]. Since then, only a few groups have evaluated Am as a replacement for the urinary tract wall. Shakeri et al. reported proper re-epithelialisation of urethrae reconstructed with Am by transitional epithelium with cytokeratin expression in a rabbit model [62]. In 2004 Koziak et al. was the first to use grafts made from Am for urethroplasty in 2 male patients [63]. Three years later, the same group demonstrated a technique for supplementation of long ureteral wall strictures with use of Am allografts [64]. Brand et al. successfully reconstructed a female urethra using autologous grafts prepared from Am [65]. The excellent integration of the implanted amnion graft with host urinary tract wall was observed after mentioned reconstructive procedures. In all cases Am reduced fibrosis and prevented stricture formation during follow-up. Due to the low mechanical resistance of Am, it required to be folded multiple times in order to obtain its carrier surface before implantation during urethroplasty and ureteral reconstruction. Electrospinning creates an opportunity to fabricate an elastic tubular-shaped biocomposite material prepared from Am and nanofibers analogous to the design presented in our study. Following this concept it would be possible to facilitate reconstruction of urethra and ureter with Am and popularise this unique biocompositie material among urologists.

The mechanical and functional evaluation of the augmented rat urinary bladders is limited by the lack of reliable research tools. Our previous study showed that urodynamic testing failed to discriminate between the differences of regenerated urinary bladders corresponding to histological regeneration [66]. In the present study, the Young’s modulus was directly measured. Elasticity of the urinary bladder wall is an active process and depends on the dynamically changeable smooth muscle contractility during the storage and emptying phase [67]. According to published studies, reconstructed urinary bladders with an applied tissue engineering approach analysed by urodynamics showed hyperactivity and improper smooth muscle tone [68,69,70,71]. This is an outcome of disordered histological structure, inadequate innervation and regulative signalling. Estimation of Young’s modulus provided data describing passive elasticity of the reconstructed bladder wall, that indirectly reflects the content of muscle and fibrotic tissue [72]. The ratio of these two components mainly determinate the stiffness of regenerated bladder wall Young modulus increased with fibrosis progression and this relationship was observed in the elasticity measurements of fibrotic organs and tissues including urinary bladder, aorta, liver, skin and pericardium [73].

Therapeutical effects reported after urinary tract reconstruction with Am are related to its supportive role during regeneration of urothelium and muscle layer. Am, a natural basement membrane of the amniotic epithelium is likely to create a favourable environment for initial urothelium re-epithelialisation and maturation [74]. Jerman et al. demonstrated that urothelium cultivated on Am was characterised with molecular and ultrastructural properties similar to that of native urothelium [75]. In our study the gradual biodegradation of PLCL nanofibers did not interrupt formation of the urothelial layer on Am. A functional urothelial layer is crucial for the regeneration process [76]. Apart from the isolation of neotissue due to the negative influence from urine, the urothelium stimulated and partially guided smooth muscle ingrowth [77]. We observed a rich distribution of smooth muscle bundles beneath the urothelium and a well developed lamina propria. At the site of adhesion between the intact and replaced bladder wall, bundles of smooth muscles were collectively observed to migrate and spread within new neotissue. Our findings support current opinions that smooth muscle development was promoted by the host’s intact bladder wall through activated fibroblasts or myofibroblasts [78]. Strong staining pattern for specific cytoskeleton protein smoothelin that is expressed exclusively by contractile smooth muscle cells indicated on newly formed muscular layers corresponding to the regenerated detrusor muscle [79, 80]. In one case, the reconstructed urinary bladder wall had the same percentage of smooth muscle surface coverage as the control urinary bladder wall (Fig 5). This finding indicated that the Am provided a distinctly favourable local environment for muscle growth. Tolg et al. demonstrated that expression of smoothelin by newly differentiated smooth muscle cells was a sign of an optimal microenvironment for regeneration [81]. Sharifiaghdas et al. reported that human smooth muscle cells cultivated on Am exhibited the same marker (actin and desmin) expression patterns as normal smooth muscle cells [82].

Our reconstructed urinary bladder wall was rich in bundles of smooth muscle cells, which formed an organised layered structure, typical for an intact urinary bladder wall. This was observed only locally and mainly close to the intact host’s bladder wall. This was similar to previous histological results where irregular arrangements of smooth muscle after urinary bladder augmentation with Am seeded with MSC were noticed [66]. Interestingly, despite the seeded MSC, the content of smooth muscle in the regenerated urinary bladder wall was lower than that of the acellular biocomposite material used in the present study.

## Conclusions

The ideal scaffold for an engineered tissue is the ECM of the target tissue in its native state. Unfortunately this requirement cannot be met in most clinical problems of reconstructive urology. Biomaterials for urethra, urinary bladder and ureter reconstruction must offer mechanical properties similar to the lower and upper wall of urinary tracts. In addition, tissue regeneration support is required. The presented bicomposite material managed to provide an microenvironment that altered the default healing response towards regeneration. Histological examination of all the explants with H&E, anti-smoothelin antibody and TRI staining confirmed that Am was able to induce formation of a new multilayered urinary bladder wall similar to a native one. Electrospun PLCL nanofibers improved the mechanical characteristic of Am without limiting its bioactivity and biocompatibility. Electrospining technology enables the creation of material with a controlled shape, size, porosity and biodegradation period. This makes it possible to adopt the physical properties of biocomposite material to meet the physiological demands of various structures within urinary tracts. The unique combination of enhanced tissue regeneration guaranteed by Am with customisation possibilities via electrospinning may lead to development of a new group of personalised biocomposite materials. The use of this technology could be adopted for the field of urethroplasty. The positive results suggest that partial urinary bladder augmentation could be taken into consideration.
